# Radicinin, a Fungal Phytotoxin as a Target-Specific Bioherbicide for Invasive Buffelgrass (*Cenchrus ciliaris*) Control

**DOI:** 10.3390/molecules24061086

**Published:** 2019-03-19

**Authors:** Marco Masi, Fabrizio Freda, Felicia Sangermano, Viola Calabrò, Alessio Cimmino, Massimo Cristofaro, Susan Meyer, Antonio Evidente

**Affiliations:** 1 Dipartimento di Scienze Chimiche, Università di Napoli Federico II, Complesso Universitario Monte S. Angelo, Via Cintia 4, 80126 Napoli, Italy; alessio.cimmino@unina.it (A.C.); evidente@unina.it (A.E.); 2 BBCA onlus, Via A. Signorelli 105, 00123 Rome, Italy; wallace_14@hotmail.com (F.F.); m.cristofaro55@gmail.com (M.C.); 3 Dipartimento di Biologia, Università di Napoli Federico II, Complesso Universitario Monte S. Angelo, Via Cintia 4, 80126 Napoli, Italy; felicia.sangermano@unina.it (F.S.); vcalabro@unina.it (V.C.); 4 ENEA C.R. Casaccia, SSPT-BIOAG-PROBIO, Via Anguillarese 301, 00123 Rome, Italy; 5 U.S. Forest Service Rocky Mountain Research Station, Shrub Sciences Laboratory, 735 North 500 East, Provo, UT 84606, USA; semeyer@xmission.com

**Keywords:** buffelgrass, foliar pathogens, *Cochliobolus australiensis*, *Pyricularia grisea*, phytotoxins, *epi*-pyriculol, radicinin

## Abstract

The fungal pathogens *Cochliobolus australiensis* and *Pyricularia grisea* have recently been isolated from diseased leaves of buffelgrass (*Cenchrus ciliaris*) in its North American range, and their ability to produce phytotoxic metabolites that could potentially be used as natural herbicides against this invasive weed was investigated. Fourteen secondary metabolites obtained from in vitro cultures of these two pathogens were tested by leaf puncture assay on the host plant at different concentrations. Radicinin and (10*S*, 11*S*)-*epi*-pyriculol proved to be the most promising compounds. Thus, their phytotoxic activity was also evaluated on non-host indigenous plants. Radicinin demonstrated high target-specific toxicity on buffelgrass, low toxicity to native plants, and no teratogenic, sub-lethal, or lethal effects on zebrafish (*Brachydanio rerio*) embryos. It is now under consideration for the development of a target-specific bioherbicide to be used against buffelgrass in natural systems where synthetic herbicides cause excessive damage to native plants.

## 1. Introduction

Buffelgrass (*Pennisetum ciliare* or *Cenchrus ciliaris*) is a perennial C_4_ bunchgrass native to the African continent, southern countries of the Mediterranean basin, and the Middle-East area [1]. Due to its fast growth, drought tolerance, resistance to heavy grazing, and forage value, it was intentionally introduced in many semi-arid regions of the world as a pasture grass [2]. In fact, *C. ciliaris* is able to settle down in harsh and different environments, tolerating temperatures up to 35 °C and heavy seasonal rains up to 2670 mm yearly [3]. Furthermore, its roots can reach 2.5 m in depth and allow the plant to reach water reserves more easily than the native plants [2]. However, it has become highly invasive in parts of its introduced range (especially in North America and Australia), negatively affecting the native vegetation, infesting roadsides and urban landscapes, and altering the wildfire regime. It is extremely competitive with native species in semi-arid ecosystems and is associated with increased wildfire [4]. Fire poses a serious and direct threat to the iconic saguaro cactus of the Sonoran Desert [5,6], and efforts to control buffelgrass there have been ongoing for many years [7]. However, effective buffelgrass control in the Sonoran Desert has been highly problematic for several reasons. It is difficult to kill with chemical herbicides, and the broad-spectrum herbicides that can sometimes be effective, e.g., glyphosate, imazapyr, also cause major damage to native species [8]. Buffelgrass is a valued forage grass throughout much of its introduced range, and any effort to develop a living biocontrol agent has been met with political resistance. At present, broad-spectrum herbicides are most commonly used for control despite the collateral damage to native plants. Hand-pulling can be effective in small areas, but it is very labor-intensive and has limited impact at a landscape scale.

Furthermore, the intensive use of these synthetic herbicides in the last fifty years had a negative environmental and ecological impact [9,10,11,12]. The accumulation of the toxins in the ecosystem and food and the rapidly evolving resistance of weeds to commercial herbicides are the two main problems. In this context, biological control has become an effective alternative to combat many weeds that invade natural systems [13,14]. The concept of weed biocontrol using pathogens or their phytotoxic metabolites has a long history [12,15], but there are few examples of pathogen-based control products that have been commercially viable [14]. These products fall into two categories: living biocontrol agents and bioherbicides based on the phytotoxins they produce [16]. Most of the available biocontrol products were developed for weed control in crops or turf and are based on naturally co-occurring pathogens that are applied in an augmentative or inundative approach [17]. These organisms by definition have narrow host ranges, which intrinsically limits their utility. They also depend on optimal conditions for disease development to achieve the epidemic levels in the field necessary for effective weed control. This can result in inconsistent performance [17]. Bioherbicides based on microbial secondary metabolites could obviate many of the problems associated with use of living pathogens for biocontrol, including the requirement for optimal conditions for disease development. To control an invasive plant species in a wildland ecosystem, the problem is essentially the reverse of the cropland weed scenario where a single crop species co-occurs with multiple weedy species. A single invader species is the target for control, and that invader occurs in a matrix of diverse native vegetation that could include a large number of potentially susceptible non-target species. Under this scenario, a target-specific bioherbicide could have great utility.

Phytotoxic metabolites vary in their degree of target-specificity. Some phytotoxins act specifically on the host species or genotype of origin (host-specific or host-selective toxins [HSTs]), usually through gene-for-gene interactions (e.g., specific toxins produced by pathotypes of *Alternaria alternata* on different hosts) [18]. A much larger group includes compounds that are toxic across many host and non-host species or genotypes, though plant species may vary in susceptibility to a particular toxin. It may not be necessary to limit the development of target-specific bioherbicides to HSTs. When new secondary metabolites are discovered, it is common practice to test and demonstrate their toxicity in bioassays on species known to be highly susceptible to a broad range of phytotoxins like, for example, tomato. However, for use of a target-specific bioherbicide in a wildland setting, a more important question is the susceptibility of co-occurring native species.

**Figure 1:**
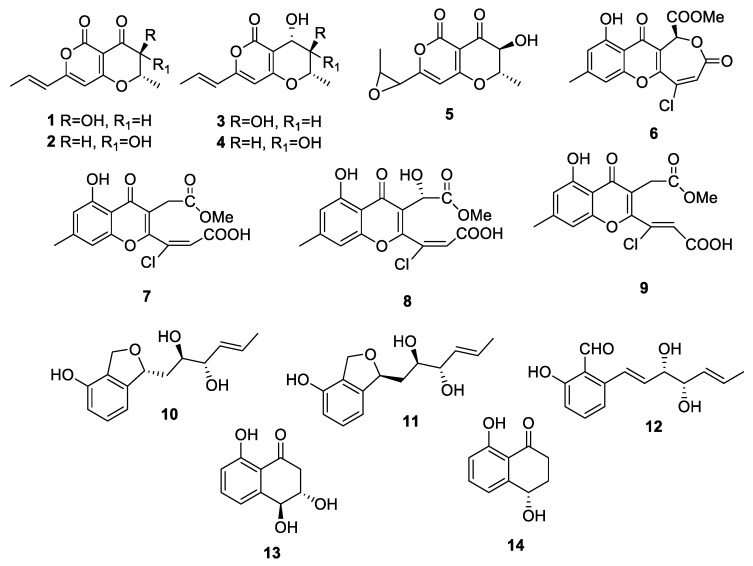
The structures of radicinin (**1**), 3-*epi*-radicinin (**2**), radicinol (**3**), 3-*epi*-radicinol (**4**), cochliotoxin (**5**), chloromonilicin (**6**), chloromonilinic acid B (**7**), chloromonilinic acid C (**8**), chloromonilinic acid D (**9**) purified from *Cochliobolus australiensis* and pyriculin A (**10**), pyriculin B (**11**), (10*S*,11*S*)-(−)-*epi*-pyriculol (**12**), *trans*-3,4-dihydro-3,4,8-trihydroxy-1(2*H*)-napthalenone (**13**), and (4*S*)-(+)-isosclerone (**14**) purified from *Pyricularia grisea*.

The research presented here explores the possibility of using a fungal plant pathogen secondary metabolite as a target-specific bioherbicide for buffelgrass (*Cenchrus ciliaris*) based on toxic compounds produced by one or more of its fungal pathogens as *Cochliobolus australiensis* and *Pyricularia grisea*. This project first involved field collection of foliar pathogens on buffelgrass in Texas and Arizona, culturing and identification of multiple strains of each of the two pathogens mentioned earlier, screening of culture filtrates from multiple strains for toxicity in seedling elongation bioassays, and selection of the best toxin-producing strains for scaled-up production in culture. Secondary metabolites contained in organic extracts from the resulting toxic culture filtrates were then isolated, purified, identified, and tested in seedling elongation bioassays to determine their toxicity to buffelgrass [19,20,21]. For one group of compounds, semi-quantitative leaf puncture bioassay results on buffelgrass and two native grasses were also reported [19]. Fourteen secondary metabolites were identified and characterized, five from *P. grisea* [21] and nine from *C. australiensis* [19,20] (**1**–**14**, Table 1, Figure 1). In the present work, these fourteen compounds were systematically tested in quantitative leaf puncture bioassays against buffelgrass at two concentrations. The most toxic of these compounds were then tested at these two concentrations on a panel of six native southwest desert species as a screen for target-specificity. Fish embryo acute and chronic toxicity on zebrafish (*Brachydanio rerio*) were evaluated for the most promising compound.

## 2. Results and Discussion

Compounds **1**–**9** and **10**–**14** (Figure 1, Table 1) have been isolated from PDB (Potato Dextrose Broth) cultures of *C. australiensis* and *P. grisea*, respectively, according to procedures previously published [19,20,21] and their purity checked by ^1^H NMR and LC-MS.

**Figure 2:**
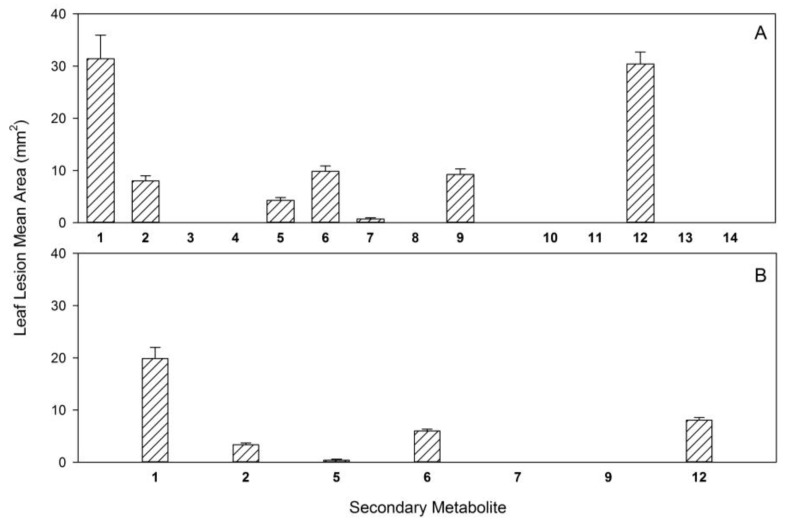
Phytotoxicity (leaf puncture bioassay) on buffelgrass (*C. ciliaris*) of secondary metabolites from *C. australiensis* and *P. grisea*: (**A**) at a concentration of 2.5 × 10^−3^ M for **1**–**14**, and (**B**) at a concentration of 10^−3^ M for **1**, **2**, **5**–**7**, **9**, and **12**.

When these metabolites were tested by buffelgrass leaf puncture assay at 2.5 × 10^−3^ M, there were highly significant differences among compounds in the necrotic lesion area (d.f. (numerator degrees of freedom) = 13, 262; F (effect mean square/error mean square) = 62.20; *p* (statistical significance) < 0.0001). Seven of the compounds (**3**, **4**, **8**, **10**, **11**, **13**, and **14**) produced no necrosis and were thus completely nontoxic to buffelgrass in this test (Figure 2A). Five compounds (**2**, **5**, **6**, **7**, and **9**) were minimally to moderately toxic while two compounds (**1** and **12**) produced lesions with areas >30 mm^2^, at least three times larger than any of the other compounds. These two compounds, radicinin (**1**) from *C. australiensis* and epi-pyriculol (**12**) from *P. grisea* were therefore highly toxic to buffelgrass leaves at this concentration. These results demonstrated that the carbonyl group at C-4 and the stereochemistry at C-3 are important structural features to impart phytotoxicity in the group of pyranopyrones (**1**–**5**). The double bond of the propenyl side chain also seemed to play a role. For the 3-chromanonacrylic acids (**7**–**9**) the *Z* stereochemistry of the double bond of the acrilic moiety appeared to be important in imparting activity. Among the hexenediols (**10**–**12**) the aldehyde group present in **12** seems to be the crucial factor in inducing phytotoxicity.

When the seven compounds (**1**, **2**, **5**, **6**, **7**, **9**, and **12**) that showed toxic activity on buffelgrass leaves at 2.5 × 10^−3^ M were tested at 10^−3^ M, significant differences among compounds were again observed (d.f. = 6.130; F = 69.39, *p* < 0.0001). Only radicinin showed high toxicity, with a reduction of approximately one-third in the lesion area relative to the lesion area at the higher concentration (Figure 2B). Compounds **2**, **5**, **6**, and **12** generally showed substantial reductions in toxicity while compounds **7** and **9** showed no activity at the lower concentration. In particular, **12** showed a reduction of approximately two-thirds in the lesion area relative to that showed at higher concentration.

**Figure 3:**
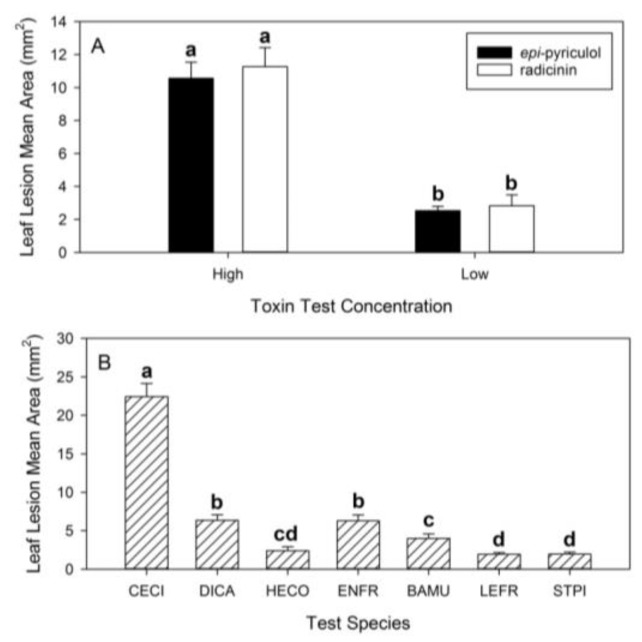
(**A**) The effects of phytotoxin identity (epi-pyriculol, **12** vs. radicinin, **1**) and concentration (low: 10^−3^ M, vs. high: 2.5 × 10^−3^ M) on leaf lesion area as measured in leaf puncture bioassays, averaged across seven test species. (**B**) The effect of test species on leaf lesion mean area, averaged across two toxins at two concentrations. (CECI = *Cenchrus ciliaris*, DICA = *Digitaria californica*, HECO = *Heteropogon contortus*, ENFR = *Encelia frutescens*, BAMU = *Baileya radiata*, LEFR = *Lepidium fremontii*, STPI = *Stanleya pinnata*). See Table 3 for statistical analysis. Error bars represent standard error of the mean. For each response variable, columns headed by the same letter are not significantly different at *p* < 0.05 based on mean separation from analysis of variance on log-transformed data.

The two most toxic compounds, radicinin (**1**) and epi-pyriculol (**12**), from the buffelgrass leaf puncture bioassays were selected for testing against the non-target host panel (Table 2). These two compounds did not differ overall in toxicity in this experiment when averaged across species (Table 3; Figure 3A). The lesion area at the lower concentration averaged 25% of lesion area at the higher concentration, and this effect of concentration did not differ between the two compounds.

There was also a highly significant effect of test species on the lesion area averaged across compounds and concentrations (Figure 3B). Buffelgrass showed necrotic lesions that were four times larger on average than the lesions on leaves of native species. The native species differed significantly in lesion areas among themselves, but the differences were relatively minor.

**Figure 4:**
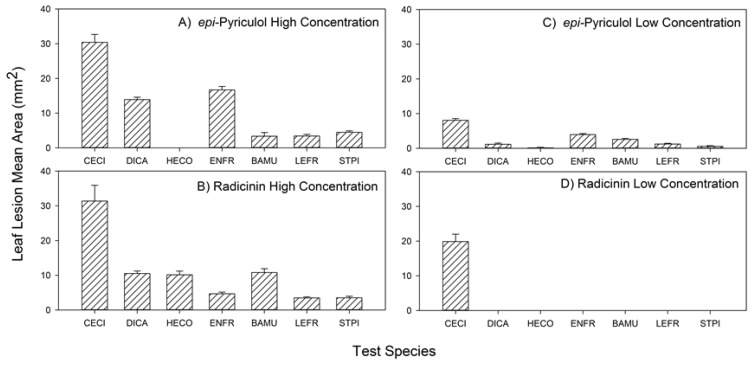
The three-way interaction among phytotoxin identity (epi-pyriculol, **12** vs. radicinin, **1**), concentration (low: 10^−3^ M vs. high: 2.5 × 10^−3^ M), and test species (CECI = *Cenchrus ciliaris*, DICA = *Digitaria californica*, HECO = *Heteropogon contortus*, ENFR = *Encelia frutescens*, BAMU = *Baileya radiata*, LEFR = *Lepidium fremontii*, STPI = *Stanleya pinnata*) for the response variable leaf lesion area. See Table 3 for statistical analysis.

There was a highly significant three-way interaction among species, toxin identity, and concentration in this experiment (Table 3). Buffelgrass was always significantly more negatively impacted (significantly larger lesion area) than any native species regardless of which toxin or concentration is considered (Figure 4). At the higher concentration, *epi*-pyriculol and radicinin showed a similar pattern and were substantially more toxic to buffelgrass than to any native species, averaging 4–6 times larger necrotic lesions on buffelgrass (Figure 4A,B). At the lower concentration, however, the two toxins had markedly different effects (Figure 4C,D). The compound *epi*-pyriculol showed reduced toxicity on buffelgrass at this concentration as mentioned earlier and as a consequence was not dramatically less toxic on native species than on buffelgrass (Figure 4C). In contrast, the target-specific effect of radicinin at this lower concentration was strongly evident. It was able to maintain high toxicity on buffelgrass but had no toxic effect on all the other species (Figure 4D).

The phytotoxic compound radicinin, isolated from the culture medium of a strain of *C. australiensis* from buffelgrass, exhibited a high degree of target-specificity on the host of origin. Radicinin is a compound known to be produced by other species of *Cochliobolus* [22] and also by other related fungi, e.g., *Alternaria chrysanthemi* [23]. It therefore belongs to the large class of fungal phytotoxins with general effects rather than representing a host-specific toxin. Its target-specificity appears to be more a function of differential resistance to the effect of the toxin, with native species included in the panel being much less sensitive than buffelgrass. The differential effect was most marked at a concentration of 10^−3^ M, supporting this interpretation. In an earlier leaf puncture bioassay study using a much less precise semi-quantitative scoring protocol, the two native grass species (*Digitaria californica* and *Heteropogon contortus*) included here were tested along with buffelgrass at the higher concentration (5 × 10^−3^ M) [15]. In this study, radicinin as well as 3-*epi*-radicinin and cochliotoxin were highly toxic to all three grass species. In a parallel test at 2.5 × 10^−3^ M, the results obtained were similar to those reported for that concentration here, with radicinin being significantly more toxic than the other two compounds. The ability of radicinin to negatively impact buffelgrass leaves even at concentrations that have no effect on native species makes it a promising candidate for bioherbicide development.

The native species test panel represented a diversity of growth habits and phylogenetic affiliations, but was limited in scope (Table 2). It is possible that some common native species that co-occur with buffelgrass in the Sonoran Desert are much more sensitive to radicinin than those included in this test panel. Toxicity screening at multiple concentrations on a wider array of native non-target species is a necessary next step in the development of radicinin as a bioherbicide for buffelgrass. Some of the compounds in Table 1 exhibited high toxicity in earlier seedling bioassay tests but were largely nontoxic on buffelgrass leaves in the present study. For example, all three chloromonolinic acids delayed seed germination and severely suppressed both radicle and coleoptile development [20]. This suggests that it may be possible to develop a bioherbicide for buffelgrass that contains multiple phytotoxic constituents with different modes of action, e.g., radicinin to damage or kill mature plants and chloromonolinic acid to act as a pre-emergent. Potential pre-emergent bioherbicides would also need careful screening on seeds of non-target species to determine their target-specificity.

**Figure 5:**
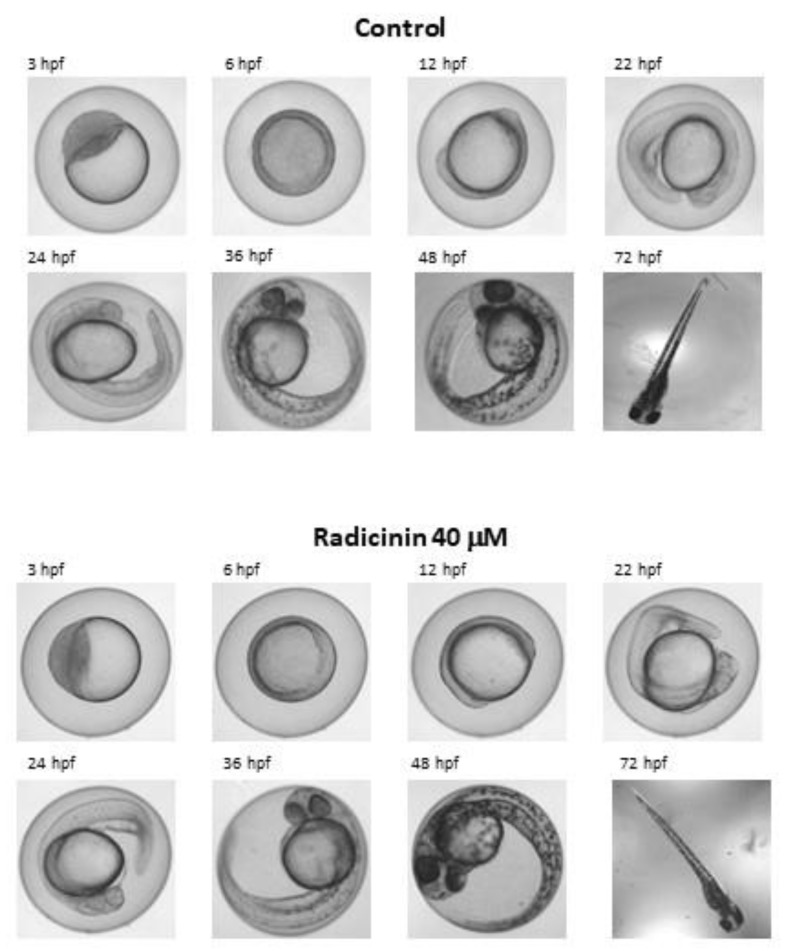
A representative panel showing normal development of *Brachydanio rerio* treated with 40 µM radicinin for the indicated times in comparison with the control (DMSO (dimethyl sulfoxide) 0.4%). Hps, hours post fertilization.

Considering the potential of radicinin to develop a target-specific herbicide, a preliminary evaluation of its ecotoxicological activity was performed using a fish embryo test (FET). This test is a highly predictive and sensitive tool for accurate and efficient evaluation of potential risks to human and ecological receptors [24]. Given its high number of eggs per spawning act, rapid development, the perfect transparency of its eggs, and the immense body of already existing information on zebrafish development, *B. rerio* seems to be the first choice for routine embryo toxicity testing. A total of ten eggs for each treatment level (1, 5, 10, and 40 µM radicinin) were tested. Lethal, sub-lethal, and teratogenic endpoints were recorded by microscopy within 72 hpf (hours post fertilization). The entire sample of embryos in the control treatment showed neither sub-lethal nor lethal effects. Compound **1** does not seem to interfere at any stage of development, as radicinin-treated embryos were undistinguishable from the controls at all concentrations tested. A representative panel showing the normal development of *B. rerio* treated with 40 µM radicinin in comparison with the control is reported in Figure 5.

## 3. Materials and Methods

### 3.1. General Experimental Procedures

^1^H-NMR spectra were recorded at 500 or 400 MHz in CDCl_3_ or CD_3_OD on a Varian (Varian, Palo Alto, CA, USA) or Bruker (Karlsruhe, Germany) spectrometer. The same solvent was used as internal standard. ESIMS (ElectroSpray Ionization Mass Spectroscopy) spectra were recorded on an Agilent 6120 Quadrupole LC/MS instrument (Agilent Technologies, Milan, Italy). Analytical and preparative TLC (thin layer chromatography) were performed on silica gel (Kieselgel 60, F254, 0.25 and 0.5 mm, respectively) plates. The spots were visualized by exposure to UV radiation (253 nm) or by spraying first with 10% H_2_SO_4_ in MeOH and then with 5% phosphomolybdic acid in EtOH, followed by heating at 110 °C for 10 min. Column chromatography was performed using silica gel (Merck, Kieselgel, Darmstadt, Germany, 60, 0.06–0.200 mm).

### 3.2. Fungal Strains

The *Pyricularia grisea* (SNM22) strain used in this study was isolated from diseased buffelgrass tissue collected in Saguaro National Monument, Arizona, USA, in autumn 2014 as well as *Cochliobolus australiensis* (LJ-4B) was isolated from the same infected plant collected near La Joya, Hidalgo County in south Texas, USA, in September 2014. Both isolates are maintained on potato dextrose agar (PDA, Fluka, Sigma-Aldrich Chemic GmbH, Buchs, Switzerland) and stored at 4 °C in the strain collection of S. Meyer at the USFS RMRS Shrub Sciences Laboratory, Provo UT, USA.

### 3.3. Isolation of Fungal Metabolites

The fungal metabolites **1**–**14** (Figure 1, Table 1) were isolated from in vitro PDB cultures of *C. australiensis* (compounds **1**–**9**) and *P. grisea* (compounds **10**–**14**) according to previously published procedures and compared to the standard compounds by TLC carried out using different solvent systems [19,20,21]. Compound purity was >98%, as checked by ^1^H-NMR and LC-MS.

### 3.4. Leaf Puncture Bioassays

Leaves of buffelgrass (*Cenchrus ciliaris*) and six non-target native species (*Digitaria californica*, *Heteropogon contortus*, *Baileya multiradiata*, *Stanleya pinnata*, *Encelia farinose*, and *Lepidium fremontii*) were included in these bioassays. Non-target natives were typical native southwest desert species with a range of growth forms and taxonomic affiliations (Table 2). The fourteen natural compounds were first assayed at 2.5 × 10^−3^ M for phytotoxicity against buffelgrass, and those active at this concentration were also bioassayed at 10^−3^ M. For the test of phytotoxicity on non-target species, the two compounds most active against buffelgrass at 2.5 × 10^−3^ M were included at both concentrations. The objectives were to first identify the compounds that could do the most damage against buffelgrass leaves, then to determine how much damage these compounds caused on leaves of non-target species. Compounds were first dissolved in MeOH (final concentration: 4%). Stock solutions at the two concentrations using sterile distilled water were then prepared for each compound. For each plant species × compound × concentration treatment combination included in a test, 24 × 3–cm leaf sections were used. An incision of ca. 3 mm was made on the adaxial surface of each leaf section with an insulin needle. The leaf sections were placed in groups of six on the surface of a water-saturated filter paper in each of four petri dishes. Five leaf sections in each petri dish were tested with the solution containing the compound while one leaf section was used as a negative control (4% MeOH only). A droplet (10 μL) of the appropriate solution was applied over each needle incision using a micropipette. The dishes were sealed with parafilm and incubated at 24 °C for 3 days in a temperature-regulated chamber under a photoperiod of 14–10 h (light/dark). After 3 days of treatment, necrotic lesion development was evaluated by removing the petri dish cover, placing a glass disc on the leaf sections to flatten them into a single plane, and photographing each dish with its leaf sections. Each acquired image was then analyzed with the software ImageJ (National Institutes of Health, Bethesda, MD, USA) to measure the necrotic area caused by the solution.

### 3.5. Statistical Analyses

The quantitative necrotic lesion data from each of the three experiments were analyzed using SAS Proc Mixed for a randomized block design with repeated subsampling. Groups of petri dishes with one dish per treatment combination were considered blocks, and leaf sections within petri dishes were considered replicate subsamples. The negative controls never showed lesion development and were excluded from the analyses. The first experiment had fourteen treatments, namely the fourteen natural compounds tested against buffelgrass at the higher concentration, while the second experiment included results from the seven compounds that showed some toxicity on buffelgrass at the higher concentration when tested at the lower concentration. The third experiment had a factorial design with the two most toxic compounds, two concentrations, and seven species, including the six non-target species (Table 3) and buffelgrass.

### 3.6. Fish Embryo Toxicity (FET) Test

Zebrafish embryos for the test were obtained from an in-house culture at the Karlsruhe Institute of Technology (Karlsruhe, Germany). The toxicity test was carried out against zebrafish embryos of wild-type Tübingen strain. The fish were crossed in the morning and the eggs were collected one hour later. The fertilized eggs were then treated with increasing doses of radicinin (**1**) from 1 to 40 μM in fish water. Radicinin was dissolved in 100% DMSO and diluted in fish water to reach the final concentration. Each fertilized egg was placed in a well and submerged in a final volume of 250 μL of radicinin in fish water. Two types of controls were made: one in 0.4% DMSO and the other in simple fish water. Embryos were observed under an optical microscope to evaluate the progress of embryonic development until 72 hpf.

## 4. Conclusions

The work reported here represents a promising next step in the effort to develop a target-specific bioherbicide for buffelgrass, but there are still many hurdles to be overcome in this process. First, there is a need to obtain much larger quantities of radicinin, either through direct synthesis or through scaling up the culture and developing an environmentally friendly extraction process using green chemistry, in order to engage in testing at the whole-plant level as well as under field conditions. Furthermore, radicinin has limited solubility in water, making development of a delivery system a challenging task. If these hurdles can be overcome and results continue to be promising, a future task will be a full ecotoxicological characterization to ascertain the fate and effect of this compound in the field environment. However, the absence of radicinin toxicity on zebrafish is an encouraging first result.

This study confirms that the concept of using secondary metabolites obtained from plant pathogens isolated from target species as target-specific bioherbicides for invasive species control merits further investigation. It may represent an approach with broad application.

## Figures and Tables

**Table 1:** Candidate secondary metabolites isolated from strains of two foliar fungal pathogens on buffelgrass (*Cenchrus ciliaris*) and included in the leaf puncture bioassay screening.

Compound	Seedling Bioassay Toxicity ^1^	Reference
From *Cochliobolus australiensis*		
radicinin (**1**)	high	[19]
3-*epi*-radicinin (**2**)	high	[19]
radicinol (**3**)	nil	[19]
3-*epi*-radicinol (**4**)	nil	[19]
cochliotoxin (**5**)	high	[19]
chloromonilicin (**6**)	not tested	[20]
chloromonilinic acid B (**7**)	high	[20]
chloromonilinic acid C (**8**)	high	[20]
chloromonilinic acid D (**9**)	high	[20]
From *Pyricularia grisea*		
pyriculin A (**10**)	low	[21]
pyriculin B (**11**)	nil	[21]
(10*S*, 11*S*)-(−)-*epi*-pyriculol (**12**)	high	[21]
*trans*-3,4-dihydro-3,4,8-trihydroxy-1(*2H*)-naphtalenone (**13**)	nil	[21]
(4*S*)-(+)-isosclerone (**14**)	nil	[21]

^1^ For details on seedling bioassay procedure and on the relative results, see cited publications [19,20,21].

**Table 2:** Information on six non-target native species used in the phytotoxin test panel.

Species	Family	Common Name	Growth Form	Primary Geographic Distribution
*Digitaria californica*	Poaceae	Arizona Cottontop	C4 perennial grass	Sonoran and Chihuahuan Deserts
*Heteropogon contortus*	Poaceae	Tanglehead	C4 perennial grass	Sonoran and Chihuahuan Deserts
*Baileya multiradiata*	Asteraceae	Desert Marigold	Perennial dicot	Mojave, Sonoran and Chihuahuan Deserts
*Stanleya pinnata*	Brassicaceae	Prince’s Plume	Perennial dicot	Western North America (widespread)
*Encelia frutescens*	Asteraceae	Brittlebush	Shrub	Mojave, Sonoran Deserts
*Lepidium fremontii*	Brassicaceae	Fremont Pepperbush	Shrub	Mojave Desert

**Table 3:** Mixed model analysis of variance (SAS Proc Mixed) for the dependent variable leaf lesion area in response to leaf puncture bioassay with two fungal toxins at two concentrations onto leaves of the target species buffelgrass and six non-target native species.

Type 3 Tests of Fixed Effects				
Effect	Num DF ^1^	Den DF ^2^	F Value ^3^	*p*-Value ^4^
species	6	498	150.49	<0001
toxin	1	498	0.48	0.4876
species × toxin	6	498	16.74	<0001
concentration	1	498	295.74	<0001
species × concentration	6	498	17.84	<0001
toxin × concentration	1	498	0.00	0.9616
species × toxin × concentration	6	498	11.68	<0001

**^1^** Num (effect) d.f. = numerator degrees of freedom, **^2^** Den d.f. = denominator (error) degrees of freedom; **^3^** F-value = effect mean square/error mean square; **^4^** *p*-value = statistical significance, probability that the observed F-value could occur by chance.

## References

[1] Abella S.R., Chiquoine L.P., Backer D.M. (2012). Ecological characteristics of sites invaded by buffelgrass (*Pennisetum ciliare*). Invasive Plant Sci. Manag..

[2] Marshall V.M., Lewis M.M., Ostendorf B. (2012). Buffelgrass (*Cenchrus ciliaris*) as an invader and threat to biodiversity in arid environments: A review. J. Arid. Environ..

[3] De La Barrera E., Castellanos A.E. (2007). High temperature effects on gas exchange for the invasive buffelgrass (*Pennisetum ciliare* L.). Weed Biol. Manag..

[4] Burquez-Montijo A.M., Miller M.E., Yrizar A.M., Tellman B., Tellman B. (2002). Mexican grasslands, Thornscrub, and the transformation of the Sonoran Desert by invasive exotic buffelgrass (*Pennisetum ciliare*). Invasive Exotic Species in the Sonoran Region.

[5] Stevens J., Falk D.A. (2009). Can buffelgrass invasions be controlled in the American Southwest? Using invasion ecology theory to understand buffelgrass success and develop comprehensive restoration and management. Ecol. Restor..

[6] Olsson A.D., Betancourt J.L., Crimmins M.A., Marsh S.E. (2012). Constancy of local spread rates for buffelgrass (*Pennisetum ciliare* L.) in the Arizona Upland of the Sonoran Desert. J. Arid. Environ..

[7] Rogstad A., Rogstad A. (2008). The Buffelgrass Strategic Plan.

[8] Bean T.M. (2014). Tools for Improved Management of Buffelgrass in the Sonoran Desert. Ph.D. Dissertation.

[9] Singh H.P., Batish D.R., Kohli R.K., Singh H.P., Batish D.R., Kohli R.K. (2006). Handbook of Sustainable Weed Management.

[10] Dayan F.E., Duke S.O. (2014). Natural compounds as next generation herbicides. Plant Physiol..

[11] Gerwick B.C., Sparks T.C. (2014). Natural products for pest control: An analysis of their role, value and future. Pest. Man. Sci..

[12] Cimmino A., Masi M., Evidente M., Superchi S., Evidente A. (2015). Fungal phytotoxins with potential herbicidal activity: Chemical and biological characterization. Nat. Prod. Rep..

[13] Harding D.P., Raizada M.N. (2015). Controlling weeds with fungi, bacteria and viruses: A review. Front. Plant. Sci..

[14] Cordeau S., Triolet M., Wayman S., Steinberg C., Guillemin J.P. (2016). Bioherbicides: Dead in the water? A review of the existing products for integrated weed management. Crop. Prot..

[15] Charudattan R. (2001). Biological control of weeds by means of plant pathogens: Significance for integrated weed management in modern agro-ecology. BioControl.

[16] Radhakrishnan R., Alqarawi A.A., AbdAllah E.F. (2018). Bioherbicides: Current knowledge on weed control mechanism. Ecotoxicol. Environ. Saf..

[17] Bailey K.L., Abrol D.P. (2014). The bioherbicide approach to weed control using plant pathogens. Integrated Pest Management: Current Concepts and Ecological Perspective.

[18] Tsuge T., Harimoto Y., Akimitsu K., Ohtani K., Kodama M., Akagi Y., Egusa M., Yamamoto M., Otani H. (2013). Host-selective toxins produced by the plant pathogenic fungus *Alternaria alternata*. FEMS Microbiol. Rev..

[19] Masi M., Meyer S., Clement S., Cimmino A., Cristofaro M., Evidente A. (2017). Cochliotoxin, a dihydropyranopyran-4,5-dione, and its analogues produced by *Cochliobolus australiensis* display phytotoxic activity against buffelgrass (*Cenchrus ciliaris*). J. Nat. Prod..

[20] Masi M., Meyer S., Clement S., Pescitelli G., Cimmino A., Cristofaro M., Evidente A. (2017). Chloromonilinic acids C and D, two phytotoxic tetrasubstituted 3-chromanonacrylic acids isolated from *Cochliobolus australiensis* with potential herbicidal activity against buffelgrass (*Cenchrus ciliaris*). J. Nat. Prod..

[21] Masi M., Meyer S., Górecki M., Mandoli A., Di Bari L., Pescitelli G., Cimmino A., Cristofaro A., Clement S., Evidente A. (2017). Pyriculins A and B, two monosubstituted hex-4-ene-2,3-diols and other phytotoxic metabolites produced by *Pyricularia grisea* isolated from buffelgrass (*Cenchrus ciliaris*). Chirality.

[22] Aldrich T.J., Rolshausen P.E., Roper M.C., Reader J.M., Steinhaus M.J., Rapicavoli J., Vosburg D.A., Maloney K.N. (2015). Radicinin from *Cochliobolus* sp. inhibits *Xylella fastidiosa*, the causal agent of Pierce’s Disease of grapevine. Phytochemistry.

[23] Robeson D.J., Gray G.R., Strobel G.A. (1982). Production of the phytotoxins radicinin and radicinol by *Alternaria chrysanthemi*. Phytochemistry.

[24] Lahnsteiner F. (2008). The sensitivity and reproducibility of the zebrafish (*Danio rerio*) embryo test for the screening of waste water quality and for testing the toxicity of chemicals. Altern. Lab. Anim..

